# Glymphatic alterations are linked to white matter topological network dysfunction in Alzheimer's Disease

**DOI:** 10.1002/alz70856_102806

**Published:** 2025-12-24

**Authors:** Liyong Wu, Min Chu, Pedro Rosa‐Neto

**Affiliations:** ^1^ Xuanwu Hospital, Capital Medical University, Beijing, China; ^2^ McGill University Research Centre for Studies in Aging, Montreal, QC, Canada; ^3^ McGill Centre for Studies in Aging, Alzheimer's Disease Research Unit, Montreal, QC, Canada; ^4^ McGill University, Montreal, QC, Canada; ^5^ Montreal Neurological Institute, Montreal, QC, Canada

## Abstract

**Background:**

Although glymphatic dysfunction and impairment of the structural topological network dysfunction are involved in Alzheimer's Disease (AD), their associations and the mediators of the association in the AD spectrum remain unclear.

**Method:**

Glymphatic activity of the whole brain was evaluated by diffusion tensor imaging along perivascular spaces (DTI‐ALPS) and structural topological network was calculated by DTI data in participants with AD dementia (*n* = 46), mild cognitive impairment (MCI, *n* = 43), and normal controls (*n* = 167) from the TRIAD cohort. Blood was collected and plasma GFAP concentrations were analyzed.

**Result:**

Significant positive correlations were found between ALPS and global efficiency, local efficiency, and hierarchy, and a significant negative correlation with the shortest path length. Lower clustering coefficiency, shortest path length, global efficiency, local efficiency, and hierarchy when comparing the global properties between low and high ALPS groups. ALPS was associated with the nodal efficiency of the nodes in the occipital‐parietal lobes. The first principal component (PC1) of the brain regions associated with disease severity was positively correlated with ALPS (top brain regions of PC1 included bilateral superior parietal gyrus, bilateral precuneus gyrus, and superior occipital gyrus). Mediation analysis identified multiple mechanisms including Aβ, Tau, neurodegeneration, inflammation, and synaptic dysfunction as mediators between ALPS and the structural topological network. In further structural equation analysis, ALPS affects the structural topological network through Aβ to Tau and Aβ to neurodegeneration pathways.

**Conclusion:**

ALPS is associated with alterations in the structural topological network in AD. Mediators such as Aβ, Tau, neurodegeneration, inflammation, and synaptic dysfunction play significant roles in the association. The Aβ to Tau and Aβ to neurodegeneration pathways also mediate the association.

